# Differences in Food and Beverage Marketing Policies and Practices in US School Districts, by Demographic Characteristics of School Districts, 2012

**DOI:** 10.5888/pcd13.160163

**Published:** 2016-12-15

**Authors:** Caitlin L. Merlo, Shannon Michael, Nancy D. Brener, Edward Coffield, Beverly S. Kingsley, Deena Zytnick, Heidi Blanck

**Affiliations:** 1 Centers for Disease Control and Prevention, Atlanta, Georgia. Dr Kingsley is now retired; Dr Coffield is now affiliated with Hofstra University, Hempstead, New York; and Ms Zytnick is now affiliated with Tufts University, Boston, Massachusetts.

## Abstract

**Introduction:**

Foods and beverages marketed in schools are typically of poor nutritional value. School districts may adopt policies and practices to restrict marketing of unhealthful foods and to promote healthful choices. Students’ exposure to marketing practices differ by school demographics, but these differences have not yet been examined by district characteristics.

**Methods:**

We analyzed data from the 2012 School Health Policies and Practices Study to examine how food and beverage marketing and promotion policies and practices varied by district characteristics such as metropolitan status, size, and percentage of non-Hispanic white students.

**Results:**

Most practices varied significantly by district size: a higher percentage of large districts than small or medium-sized districts restricted marketing of unhealthful foods and promoted healthful options. Compared with districts whose student populations were majority (>50%) non-Hispanic white, a higher percentage of districts whose student populations were minority non-Hispanic white (≤50% non-Hispanic white) prohibited advertising of soft drinks in school buildings and on school grounds, made school meal menus available to students, and provided families with information on school nutrition programs. Compared with suburban and rural districts, a higher percentage of urban districts prohibited the sale of soft drinks on school grounds and used several practices to promote healthful options.

**Conclusion:**

Preliminary findings showing significant associations between district demographics and marketing policies and practices can be used to help states direct resources, training, and technical assistance to address food and beverage marketing and promotion to districts most in need of improvement.

## Introduction

Food and beverage marketing influences children’s purchase requests, preferences, and dietary intake and contributes to unhealthful dietary intake among American children and adolescents (1–4). Most students are exposed to at least one form of marketing during the school day (5,6). In 2009, food and beverage companies spent $149 million on marketing foods and beverages in US schools (7).

Foods and beverage marketing in schools occurs in many forms, including posters, coupons, contests, fundraisers, commercials during educational programming (eg, Channel One television), and the sale of branded foods and beverages (5–9). The advertised foods and beverages are typically of poor nutritional value (1). Advertising unhealthful foods and beverages in schools is contrary to health education curricula, creates inconsistent messages for children, adolescents, and families about healthful eating, and can promote unhealthful dietary choices (7,10,11).

School districts can help support healthful dietary behaviors by implementing policies and practices that reduce students’ exposure to marketing of unhealthful foods and beverages and promote healthful options (12). The prevalence of food and beverage marketing policies and practices in districts and schools in the United States has been described (5,13–17), and differences exist in students’ exposure to marketing practices by school demographics (5,14–16). However, differences in food marketing policies and practices have not been examined by district characteristics. Our study addresses this gap by examining whether food and beverage marketing and promotion policies and practices differ by district characteristics such as metropolitan status and size. Findings could help states understand which types of school districts are most in need of resources, training, and technical assistance to address food and beverage marketing and promotion practices, which in turn will help them meet new federal requirements for local school wellness policies (18).

## Methods

### Sample

The School Health Policies and Practices Study (SHPPS) is a national survey periodically conducted by the Centers for Disease Control and Prevention to assess school health policies and practices at the state, district, school, and classroom levels. We analyzed district-level data from the 2012 SHPPS. A detailed description of SHPPS, including methods used in 2012, is available at www.cdc.gov/healthyyouth/shpps/index.htm (17,19). Briefly, a 2-stage sample design was used to generate a nationally representative sample of public school districts in the United States. Seven questionnaires were administered in each sampled district.

Respondents identified as the most knowledgeable completed the relevant questionnaire or module via a secure data collection website or paper-and-pencil questionnaire. Response rates varied by questionnaire and module. We analyzed data from the district-level Nutrition Services questionnaire, which had a response rate of 63.0% (n = 660 districts) and from the district-level General School Environment module, which had a response rate of 60.1% (n = 630 districts).

### Measures

The Nutrition Services questionnaire asked about district practices to promote healthful foods and beverages in schools, and the General School Environment module asked about district policies to restrict marketing of unhealthful foods and beverages. The questions were categorized as either asking about restricting marketing of unhealthful foods and beverages or asking about promoting healthful foods and beverages (Box).

**Box. Questions Used in Analysis From the School Health Policies and Practices Study, 2012^a,b,c,d^**

**Policies to restrict marketing of less nutritious foods and beverages**

**Q52.** Does your district require or recommend that schools prohibit junk foods from being sold for fundraising purposes? (Require, Recommend, Neither)

**Q121.** Does your district require or recommend that schools prohibit advertisements for junk food or fast food restaurants on school property? (Require, Recommend, Neither)

**Q125.** Does your district require or recommend that schools restrict the distribution of products promoting junk food, fast food restaurants, or soft drinks to students, such as t-shirts, hats, or book covers? (Require, Recommend, Neither)

**Q130.** Are soft drink companies allowed to advertise soft drinks, such as sports drinks, soda pop, or fruit drinks that are not 100% juice (Yes/No)

**a.** In school buildings?

**b.** What about on school grounds, including on the outside of school buildings, on playing fields, or other areas of campus?

**Practices to promote more healthful foods and beverages**

**Q17.** During the past 12 months, has anyone from your district . . . (Yes/No)

**a.** Made menus available to students?

**b.** Made information available to students on the nutrition and caloric content of foods available to them?

**Q18**. During the past 12 months, has anyone from your district . . . (Yes/No)

**a.** Made menus available to families of all students?

**b.** Made information available to families of all students on the nutrition and caloric content of foods available to students?

**c.** Made information on the school nutrition services program available to families of all students?

**Q19.** During the past 12 months, has anyone from your district provided ideas to schools . . . (Yes/No)

**a.** On how to involve school nutrition services staff in classrooms?

**b.** On how to use the cafeteria as a place where students might learn about food safety, food preparation, or other nutrition-related topics?

**c.** For nutrition-related special events?

**Q32.** During the past two years, has your district provided funding for or offered professional development to nutrition services staff on . . . (Yes/No)

**d.** Using the cafeteria for nutrition education?

**j.** Strategies to improve the presentation of healthful foods in the cafeteria?

^a ^Question numbering in the table reflects the numbering used in the SHPPS questionnaires, including the district-level Nutrition Services questionnaire and the district-level General School Environment module. Both are available at www.cdc.gov/healthyyouth/shpps/questionnaires.htm.

^b ^Prevalence estimates for each variable included in this analysis are available at www.cdc.gov/healthyyouth/shpps/2012/pdf/shpps-results_2012.pdf#page=81.

^c ^For questions with response options require, recommend, or neither, require and recommend responses were combined, and all responses were reverse coded so that neither = 0 and require/recommend = 1.

^d^ Yes or no responses to Q130a and Q130b were summed and then recoded so that no = 1 (ie, did not allow soft drink companies to advertise soft drinks in school buildings and other areas of school campus) and yes = 0 (ie, allowed soft drink companies to advertise soft drinks in school buildings and/or other areas of school campus).

Data from the district questionnaires were linked with extant data on district characteristics collected in summer 2011 from MCH Strategic Data, Inc (http://mchdata.com/quality-education-data-qed/). We examined the following district characteristics: percentage of non-Hispanic white students, metropolitan status, district size, percentage of Title I students (defined by MCH as the percentage of students receiving free or reduced-price lunch), and total annual expenditures per student. We categorized these variables as follows: percentage of non-Hispanic white students as 50% or less (hereinafter referred to as minority non-Hispanic white) or as more than 50% (hereinafter referred to as majority non-Hispanic white); metropolitan status as rural, suburban (large or small town), or urban (large central city, mid-sized central city, urban fringe of central city, urban fringe of mid-sized city); district size as small (1–2,499 students), medium (2,500–9,999 students), or large (≥10,000 students); percentage of Title I students as 33% or less, more than 33% but less than 67%, or 67% or more; and total annual expenditures per student (ie, all current expenditures for a district including instructional expenditures, support services, noninstructional expenditures) as less than $8,850 (the median based on the data distribution) or $8,850 or more.

### Analysis

We used χ^2^ tests to examine whether the percentage of school districts that engaged in food and beverage marketing and promotion policies and practices varied by district characteristics. For policies and practices with 3 categories (ie, district size, metropolitan status, and percentage of Title I students), posthoc tests were conducted to determine which categories were significantly different from each other. Bonferroni α adjustments were used to account for possible increases in type 1 error.

All analyses were conducted using weighted data. To account for the complex sample design, all analyses were conducted in Sudaan version 11.0.0 (RTI International) except for posthoc tests, which were conducted in Stata version 13 (StataCorp LP).

## Results

Compared with small and medium-sized districts, a significantly higher percentage of large school districts prohibited the advertisement of junk food or fast food restaurants on school property; restricted the distribution of products promoting junk food, fast food restaurants, or soft drinks to students; and prohibited junk foods from being sold for fundraising purposes (Table 1). Additionally, a higher percentage of districts whose students populations were minority non-Hispanic white and a higher percentage of districts with $8,850 or more in total annual expenditures per student prohibited soft drink companies from advertising soft drinks in school buildings and on school grounds, compared with districts that had majority non-Hispanic white student populations and less than $8,850 in total annual expenditures per student, respectively. A higher percentage of urban districts than suburban or rural districts prohibited soft drink companies from advertising on school grounds.

**Table 1:** Percentage of School Districts With Policies That Restrict Marketing of Unhealthful Foods and Beverages, by District Demographic Characteristics, School Health Policies and Practices Study, 2012

Characteristic	Practice^a^^,^^b^				
	Prohibit Advertising Junk Food or Fast Foods	Restrict Distribution of Promotional Products	Prohibit Advertising Soft Drinks in School Buildings	Prohibit Advertising Soft Drinks on School Grounds	Prohibit Junk Food Fundraising
**Sample size, n**	602	598	536	529	633
**Total that had practice^c^**	65.9 (61.8–69.8)	57.1 (53.0–61.2)	73.5 (68.9–77.6)	66.5 (61.6–71.0)	58.4 (54.1–62.5)
**Percentage of non-Hispanic white students**					
≤50	64.9 (54.8–73.8)	54.6 (44.9–64.0)	81.9 (72.5–88.6)	77.0 (67.2–84.4)	65.1 (54.8–74.2)
>50	65.8 (61.2–70.1)	57.5 (52.8–62.0)	71.6 (66.5–76.3)	64.0 (58.5–69.1)	56.8 (52.2–61.4)
*P* value	.87	.60	.03	.01	.13
**Metro status**					
Rural	65.2 (58.9–71.0)	54.7 (48.4–60.9)	69.7 (62.1–76.3)	60.7 (52.6–68.2)	55.5 (49.3–61.6)
Suburban	60.5 (50.3–69.8)	51.3 (40.7–61.7)	72.3 (61.6–80.9)	68.2 (56.6–77.8)	55.9 (44.4–66.8)
Urban	69.1 (62.3–75.2)	61.9 (55.1–68.3)	79.2 (72.8–84.5)	73.2 (66.6–78.9)^d^	62.6 (56.2–68.5)
*P* value	.36	.15	.12	.05	.24
**District size**					
Small	53.1 (42.5–63.4)	45.9 (35.0–57.3)	72.6 (60.4–82.1)	79.2 (67.4–87.6)	42.8 (32.5–53.7)
Medium	62.0 (54.0–69.3)^e^	55.5 (47.9–62.8)	71.0 (61.5–78.9)	64.2 (54.8–72.6)	54.5 (46.4–62.4)^e^
Large	71.2 (66.0–75.8)^f^	60.8 (55.5–65.8)^f^	75.1 (69.8–79.7)	64.5 (58.7–69.9)	63.0 (57.7–68.0)^f^
*P* value	.002	.007	.40	.72	<.001
**Percentage of Title I students^g^**					
≤33	64.8 (57.5–71.4)	60.2 (52.6–67.4)	74.7 (67.6–80.7)	70.0 (62.8–76.4)	55.1 (48.4–61.6)
>33 to <67	65.6 (60.3–70.4)	54.6 (48.8–60.3)	72.4 (65.3–78.6)	63.8 (56.2–70.8)	58.8 (52.7–64.8)
≥67	68.2 (56.6–77.9)	57.4 (45.6–68.4)	72.6 (60.5–82.0)	64.3 (51.9–75.0)	66.2 (55.5–75.5)
*P* value	.87	.50	.88	.43	.20
**Total annual expenditures per student, $^h^**					
<8,850	65.7 (60.0–71.0)	58.4 (52.6–64.1)	69.1 (62.0–75.3)	59.5 (52.3–66.3)	62.0 (55.8–67.8)
≥8,850	66.2 (59.9–72.0)	55.9 (49.7–61.9)	78.2 (72.2–83.2)	73.5 (67.2–79.0)	54.1 (48.2–59.9)
*P* value	.90	.55	.04	.003	.06

a See Box for full description of each question.

b All values are percentage (95% confidence interval) unless otherwise indicated.

c Percentage of districts that had the practice.

d Indicates that likelihood ratio χ^2^ (*G* ^2^) posthoc test for urban districts vs rural districts is significant at a Bonferroni adjusted 5% α level (*P* ≤ .05).

e Indicates that likelihood ratio χ^2^ (*G* ^2^) posthoc test for differences between large and small school districts vs medium school districts is significant at a Bonferroni adjusted 5% α level (*P* ≤ .05).

f Indicates that likelihood ratio χ^2^ (*G* ^2^) for large districts vs small districts is significant at a Bonferroni adjusted 5% α level (*P* ≤ .05).

g Percentage of students receiving free or reduced-price lunch.

h The median total annual expenditures per student (ie, instructional expenditures, support services, and noninstructional expenditures).

Posthoc tests found differences between small and large districts for all 3 of the policies to restrict marketing of unhealthful foods and beverages that differed significantly by district size: a higher percentage of large districts than small districts restricted marketing. For prohibiting soft drink companies from advertising on school grounds, the posthoc tests showed significant differences between urban and rural districts.

Of the 10 promotion practices examined, a higher percentage of large school districts than small and medium-sized districts reported using all of the practices, except for using the cafeteria for nutrition education (Table 2). A lower percentage of districts with majority non-Hispanic white student populations reported making menus available to students and making information on the school nutrition services program available to families of all students, compared with districts whose student populations were minority non-Hispanic white. A higher percentage of urban districts made nutrition information available to students, made nutrition information available to students’ families, and made information on school nutrition services programs available to families of all students, compared with rural and suburban districts. A higher percentage of districts with 67% or more of Title I students made menus available to students, compared with districts with less than 67% Title 1 students. In addition, a higher percentage of districts in the middle tertile of Title 1 students provided ideas to schools on involving school nutrition service staff in classrooms, compared with districts with 33% or less Title 1 students or districts with 67% or more Title 1 students.

**Table 2:** Percentage of School Districts That Use Practices to Promote Healthful Food and Beverage Options, By District Demographic Characteristics, School Health Policies and Practices Study, 2012

Characteristic	**Practice^a^^,^^b^**				
	**Made Menus Available to Students**	**Made Nutrition Information Available to Students**	**Made Menus Available to Families**	**Made Nutrition Information Available to Families**	**Made Information on School Nutrition Services Program Available to Families**
**Sample size, n**	652	646	647	644	644
**Total^c^**	97.1 (95.7–98.2)	68.1 (64.3–71.8)	97.0 (95.3–98.1)	52.7 (48.4–56.9)	82.6 (79.0–85.8)
**Percentage of non-Hispanic white students**					
≤50%	100	70.8 (61.6–78.6)	96.6 (90.6–98.8)	58.1 (47.8–67.7)	91.9 (85.1–95.8)
>50%	96.6 (94.5–98.0)	67.7 (63.4–71.8)	97.2 (95.3–98.3)	51.7 (47.0–56.3)	81.0 (77.0–84.5)
*P* value	<.001	.53	.77	.25	<.001
**Metro status**					
Rural	96.8 (94.0–98.3)	63.3 (57.2–68.9)	96.6 (93.5–98.2)	44.9 (38.3–51.7)	78.1 (71.6–83.4)
Suburban	95.5 (84.7–98.8)	66.6 (55.4–76.3)	97.7 (91.2–99.4)	50.3 (39.6–61.0)	80.5 (71.6–87.1)
Urban	97.9 (95.1–99.1)	74.2 (68.8–78.8)^d^	97.2 (94.4–98.6)	62.6 (56.8–68.1)^d^	88.5 (83.8–91.9)^d^
*P* value	.60	.02	.82	<.001	.01
**District size**					
Small	96.2 (93.6–97.8)	63.0 (57.8–67.9)	95.9 (93.4–97.5)	47.0 (41.6–52.6)	76.5 (71.5–80.9)
Medium	98.8 (95.4–99.7)	76.4 (69.4–82.2)^e^	99.4 (95.9–99.9)^e^	59.9 (52.4–66.9)	93.2 (88.4–96.1)^e^
Large	100.0	83.5 (70.7–91.4)^f^	99.9 (99.2–100.0)^f^	75.6 (61.9–85.5)^f^	97.7 (86.5–99.6)^f^
*P* value	.002	<.001	.001	<.001	<.001
**Percentage of Title I students^g^**					
≤33%	95.7 (92.1–97.7)	69.0 (62.6–74.7)	95.3 (91.6–97.4)	52.4 (46.0–58.8)	79.7 (73.3–84.9)
>33 to <67%	97.8 (95.2–99.0)	66.0 (60.4–71.1)	98.8 (96.4–99.6)	51.9 (45.6–58.1)	84.0 (78.7–88.3)
≥67%	100.0	71.5 (60.8–80.2)	96.8 (88.5–99.2)	55.1 (42.7–66.8)	88.0 (78.8–93.6)
*P* value	.002	.56	.07	.90	.21
**Total annual expenditures per student^h^**					
<$8,850	96.7 (94.0–98.2)	71.1 (65.8–75.9)	97.2 (94.4–98.6)	54.5 (48.8–60.0)	83.5 (79.0–87.2)
≥$8,850	97.7 (95.2–98.9)	65.1 (59.5–70.3)	97.1 (94.5–98.5)	50.7 (44.6–56.9)	81.7 (76.1–86.2)
*P* value	.44	.10	.96	.37	.56
	**Ideas for Involving School Nutrition Services Staff in Classrooms**	**Ideas for Using the Cafeteria for Nutrition Education**	**Information on School Nutrition Services Program Available to Families**	**Professional Development on Using the Cafeteria for Nutrition Education**	**Professional Development on Presenting Healthful Foods in the Cafeteria**
**Sample size, n**	646	641	644	628	632
**Total^c^**	47.2 (43.0–51.3)	47.3 (43.2–51.4)	52.4 (48.3–56.4)	48.1 (43.8–52.3)	71.4 (67.6–74.9)
**Percentage of non-Hispanic white students**					
≤50%	54.4 (45.2–63.3)	49.6 (40.4–58.8)	57.7 (48.0–66.8)	51.8 (42.2–61.3)	70.9 (62.4–78.2)
>50%	45.9 (41.3–50.6)	46.8 (42.2–51.4)	51.4 (46.9 −55.9)	47.3 (42.5–52.1)	71.8 (67.5–75.7)
*P* value	.10	.59	.24	.41	.85
**Metro status**					
Rural	47.6 (41.2–54.2)	46.0 (39.8–52.3)	47.5 (41.2–53.9)	47.4 (40.7–54.2)	70.6 (64.6–76.0)
Suburban	51.6 (41.8–61.3)	52.6 (42.4–62.6)	52.5 (41.4–63.3)	49.6 (38.2–61.0)	68.0 (57.8–76.6)
Urban	44.2 (38.2–50.4)	46.2 (39.9–52.7)	57.5 (51.4–63.5)	47.9 (41.6–54.3)	74.0 (67.8–79.4)
*P* value	.43	.52	.08	.95	.85
**District size**					
Small	42.8 (37.5–48.3)	44.5 (39.4–49.7)	43.7 (38.7–48.9)	47.3 (41.8–52.8)	68.7 (63.7–73.4)
Medium	53.0 (45.5–60.4)	47.9 (40.5–55.4)	64.8 (57.5–71.5)^e^	46.1 (38.4–54.0)	74.0 (67.0–80.0)
Large	67.5 (53.6–78.9)	68.2 (54.2–79.5)^f^	83.2 (70.7–91.1)^f^	63.8 (49.7–75.8)	85.2 (73.4–92.3)^f^
*P* value	.002	.01	<.001	.07	.02
**Percentage of Title I students^g^**					
≤33%	39.3 (32.8–46.1)	42.6 (35.9–49.5)	47.2 (40.5–53.9)	43.7 (37.2–50.5)	73.0 (66.8–78.4)
>33% to <67%	53.1 (47.1–59.0)^i^	51.4 (45.7–57.1)	54.5 (48.5–60.4)	51.3 (44.9–57.7)	71.1 (65.3–76.2)
≥67%	51.1 (40.0–62.0)	46.6 (35.0–58.5)	59.0 (48.4–68.9)	48.6 (39.1–58.3)	69.4 (58.4–78.6)
*P* value	.01	.15	.12	.26	.80
**Total annual expenditures per student^h^**					
<$8,850	50.0 (44.1–55.9)	48.9 (43.2–54.6)	52.4 (46.7–58.0)	50.4 (44.5–56.3)	71.0 (65.5–75.9)
≥$8,850	44.8 (39.1–50.6)	45.4 (39.6–51.2)	52.2 (46.5–58.0)	45.9 (39.9–51.9)	71.8 (66.7–76.3)
*P* value	.21	.34	.97	.28	.82

Abbreviations: CI, confidence interval; NHANES, National Health and Nutrition Examination Survey.

a See Box for full description of each question.

b All values are percentage (95% confidence interval) unless otherwise indicated.

c Percentage of districts that had the practice.

d Indicates that likelihood ratio χ^2^ (*G* ^2^) posthoc test for urban districts vs rural districts is significant at a Bonferroni adjusted 5% α level (*P* ≤ .05).

e Indicates that likelihood ratio χ^2^ (*G* ^2^) posthoc test for differences between large and small school districts vs medium school districts is significant at a Bonferroni adjusted 5% α level (*P* ≤ .05).

f Indicates that likelihood ratio χ^2^ (*G* ^2^) for large districts vs small districts is significant at a Bonferroni adjusted 5% α level (*P* ≤ .05).

g Percentage of students receiving free or reduced-price lunch.

h The median total annual expenditures per student (ie, instructional expenditures, support services, noninstructional expenditures).

i Indicates that likelihood ratio χ^2^ (*G* ^2^) posthoc test for differences between districts with ≥67% of students qualifying for Title 1 and districts with ≤33% students qualifying for Title 1 vs districts with >33% to <67% of students qualifying for Title 1 is significant at a Bonferroni adjusted 5% α level (*P* ≤ .05).

Of the 9 practices to promote healthful food and beverage options that significantly differed by district size, posthoc tests indicated that 7 practices differed significantly between small and large districts. Small and large districts also differed from medium-sized districts for 4 of these practices. For all 3 practices that significantly differed by metropolitan status, posthoc tests indicated that these differences were between urban districts and rural districts. Additionally, the percentage of districts providing schools with ideas for involving school nutrition services staff in classrooms significantly differed for districts with more than 33% to less than 67% of Title I students compared with districts with 33% or less or 67% or more of students qualifying for Title I.

## Discussion

To our knowledge, this analysis is the first to illustrate that policies and practices intended to restrict marketing of unhealthful foods and beverages or promote healthful items vary by district demographics. We found that policies and practices varied by all of the demographic characteristics examined: the percentage of non-Hispanic white students, metropolitan status, district size, percentage of Title I students, and total annual expenditures per student.

Across the 15 policies and practices examined in our study, we found significant differences for 12 of them by district size, with a higher percentage of large districts using both policies and practices to restrict marketing of unhealthful foods and beverages and promote healthful options. One possible explanation for these findings is that food and beverage companies might see large districts as a stronger advertising market and approach them more frequently with marketing opportunities. Large districts might respond to these approaches by adopting policies to limit marketing.

Another possible explanation is that district size could be a proxy variable for a district’s overall infrastructure to support health policies and practices. For example, large school districts may be more likely than small districts to have a school health coordinator, an active district wellness committee, or a strong local school wellness policy with provisions on marketing and promotion of foods and beverages. Although in 2012–2013 only 22% of school districts had policies that required or recommended restricting the marketing of unhealthful items on school grounds (20), an analysis published in 2009 of local school wellness policies found that larger school districts had stronger wellness policies overall (21). Additionally, small districts may have fewer resources than large districts to address food marketing and promotion, and large school districts may provide more training and professional development opportunities for staff on school health topics, including strategies to promote more healthful foods and beverages. One study of school practices found that small schools had lower odds of having a school health council than large schools (22). School supports, such as a school health coordinator, the presence of a school health council, and a school health council with a diverse membership are associated with more implementation of strategies to promote healthful options (15).

Four of the policies and practices that we examined differed by metropolitan status, with more urban districts engaging in both restricting policies and promoting practices than suburban and rural districts. This finding is similar to the findings of a study that used data from 2008 to 2012 on secondary schools in Minnesota and found that city schools were more likely than rural schools to implement policies and practices to support a healthful school nutrition environment, including banning advertisements of unhealthful foods and beverages (16). That study also found that the prevalence of these practices decreased over time in city schools, indicating that schools may need additional support to sustain marketing bans.

A higher percentage of districts with a minority non-Hispanic white student population prohibited advertising soft drinks in school buildings and on school grounds, compared with districts with a majority non-Hispanic white student population. A study of food and beverage marketing in secondary schools using data from 2007 to 2012 also found that students’ exposure to different forms of marketing varied by the racial/ethnic composition of the schools’ student body. Although that study examined different marketing practices than those addressed in our study, the findings showed greater exposure to certain forms of marketing among students attending schools with a predominantly non-Hispanic white student body (5). The authors of that study stated that these findings may have resulted from the lower prevalence of vending machines in majority African American schools, thereby decreasing prevalence of related marketing practices (eg, exclusive beverage contracts).

The US Department of Agriculture’s final rule for local school wellness policies requires school districts to include language in the district wellness policy that prohibits the marketing and promotion of foods and beverages that do not meet or exceed the minimum federal nutrition standards for all foods sold at school (ie, Smart Snacks in School nutrition standards) (18,23). Additionally, the final rule requires districts to include a goal in the wellness policy for nutrition promotion, which may include strategies to encourage students to make healthful choices, such as placing fruit near the cash register, where students can easily see it; using attractive displays (eg, baskets) for whole fruit; offering at least 2 vegetable options each day for lunch; and placing unflavored milk in front of other beverages options (24–26).

Although these requirements will likely result in more districts adopting food marketing policies and practices, states play an important role in helping districts and schools understand local school wellness policy requirements by sharing model policy language, providing guidance on evidence-based strategies to meet or exceed federal requirements, and ensuring that policies are implemented in schools (27). The results of our study and other recent studies (5,14–16) may help inform which types of school districts and schools (eg, small, rural) to prioritize with this assistance, including guidance on the types of structures (eg, school health coordinator, school health council) that should be in place to implement health policies and practices. State agencies can provide training and technical assistance to school districts and schools on establishing a school health council with broad school and community representation, identifying food and beverage marketing in the school setting through direct observation, and using evidence-based strategies to minimize or eliminate unhealthful food and beverage marketing and better promote healthful options.

To our knowledge, our study is the first study to examine policies and practices to both restrict marketing of unhealthful foods and promote healthful choices in a nationally representative sample of school districts. However, this study also has several limitations. First, SHPPS data are self-reported; district policies and practices were not verified through other sources. Additionally, although results were weighted to adjust for nonresponse, differences between responding districts and nonresponding districts were not examined and could potentially bias the results. SHPPS is a cross-sectional study; therefore causality between district characteristics and practices cannot be inferred. Finally, contextual factors that may influence district policies and practices (eg, district administration support) were not included in this study.

Additional research is warranted to better understand the extent of marketing and promotion policies and practices and associations between district characteristics and such practices. For example, direct observations (28,29) or photographs in schools may help districts better quantify and describe differences in marketing and promotion practices that are not identified through surveys. This information could help districts develop wellness policy goals for marketing that reflect the needs of the schools in the district. Qualitative research including focus groups and key informant interviews could help clarify why certain practices are more feasible for districts to implement and how contextual factors such as district support for school health issues can affect implementation. Additionally, understanding how state guidance and technical assistance to school districts affects district policies and practices could help states prioritize resources and training efforts.

Addressing food and beverage marketing and promotion is a critical step in creating healthful school nutrition environments with consistent messages about good nutrition (30). Further analysis of the data from Minnesota from 2008 to 2012 showed that although the percentage of schools using healthful food and beverage promotion strategies increased significantly, the percentage of schools banning advertising of unhealthful items did not increase (15). National data also showed no significant improvements in many practices related to advertising of unhealthful foods and beverages (13,31). These data could indicate that schools may find it easier to promote healthful options than to prohibit the marketing of unhealthful foods and beverages. Understanding how marketing and promotion policies and practices differ in schools districts and schools can guide training and technical assistance efforts to help them meet or exceed new requirements for local school wellness policies.
